# Nd:YAG/Er:YAG dual laser vs. topical steroid to treat vulvar lichen sclerosus: study protocol of a randomized controlled trial

**DOI:** 10.1007/s00404-023-07055-z

**Published:** 2023-05-05

**Authors:** Volker Viereck, Marianne Gamper, Sigrid Regauer, Claudia Walser, Irena Zivanovic

**Affiliations:** 1 Department of Gynecology and Obstetrics, Cantonal Hospital Frauenfeld Postfach, 8501, Frauenfeld, Switzerland; 2 Institute of Pathology, Medical University Graz, Graz, Austria

**Keywords:** Neodymium:YAG laser, Erbium:YAG laser, RCT, Gold standard, Physician-administered clinical lichen sclerosus score

## Abstract

**Purpose:**

Vulvar lichen sclerosus (LS) is a chronic debilitating inflammatory skin disease. Today, the gold standard is a life-long topical steroid treatment. Alternative options are highly desired. We present a study protocol of a prospective, randomized, active-controlled, investigator-initiated clinical trial comparing a novel non-invasive dual Nd:YAG/Er:YAG laser therapy with the gold standard for the management of LS.

**Methods:**

We recruited 66 patients, 44 in the laser arm and 22 in the steroid arm. Patients with a physician-administered clinical LS score ≥ 4 were included. Participants received either four laser treatments 1–2 months apart, or 6 months of topical steroid application. Follow-ups were planned at 6, 12, and 24 months. The primary outcome looks at the efficacy of the laser treatment at the 6-month follow-up. Secondary outcomes look at comparisons between baseline and follow-ups within the laser or the steroid arm, and comparisons between laser vs. steroid arm. Objective (LS score, histopathology, photo documentation) and subjective (Vulvovaginal Symptoms Questionnaire, symptom VAS score, patient satisfaction) measurements, tolerability, and adverse events are evaluated.

**Conclusion:**

The findings of this trial have the potential to offer a novel treatment option for LS. The standardized Nd:YAG/Er:YAG laser settings and the treatment regime are presented in this paper.

**Clinical trial identification number:**

NCT03926299.

## What does this study add to the clinical work

**Table:** 

Presentation of a novel non-invasive double laser therapy to treat vulvar lichen sclerosus. In a randomized controlled trial with a follow-up of two years, the new laser therapy is evaluated as an alternative to the gold standard treatment with topical steroid.	

## Introduction

Lichen sclerosus (LS) is a chronic inflammatory skin disease most commonly found in adult women. It is almost exclusively restricted to the anogenital region [1]. The incidence rate is highest in postmenopausal women (24–53 of 100,000 per year) and the prevalence is 2–7% [2]. The typical symptom is vulvar itching, further symptoms include vulvar burning or stinging, vulvar pain, and dyspareunia [2], with a high impact on quality of life. A significant percentage of cases can also be asymptomatic (15–40%) [3], and the diagnosis may be delayed. LS is a progressive autoimmune disease with an intense inflammation process leading to destruction of the loose connective tissue and to clear macroscopic changes of the vulvar anatomy with white atrophic plaques, depigmentation, erosions, hyperkeratosis, fissures, agglutination with labial resorption, clitoral phimosis and introital stenosis [4]. When untreated, the lifetime risk for a vulvar carcinoma is 5% [5].

The application of topical steroids is the gold standard for treating female genital LS [6]. There is no standardized treatment regime; however, the potent steroid clobetasol propionate, 0.05% ointment or cream once or twice daily for the first few months with a reduction of application frequency for the subsequent months is recommended to avoid skin thinning. A maintenance treatment prevents severe relapses [6] and tumor progression [5]. Treatment improves symptoms and can reverse early signs. However, severe architectural changes are irreversible [6]. Additional moisturizers improve symptoms.

Side effects of steroid treatment include skin atrophy, superimposed infections and adrenal insufficiency, and furthermore, a strict compliance with the treatment regime is required [7]. Advanced stages of LS with an epithelial hyperkeratosis and a band-shaped subepithelial sclerosis can be refractory to standard steroid treatment, most likely because the topically applied medication cannot penetrate the broad tissue layers [8, 9]. There is a great interest for alternative therapies among patients and practitioners.

Laser therapy of the vulva might be such an alternative, potentially even circumventing some of the disadvantages of steroid therapy. A recent review on laser treatment for LS identified three randomized controlled trials (RCTs), three cohort studies, and four case reports [10]. In nine studies including two RCTs [7, 11], the fractional ablative CO_2_ laser was used, one RCT used the non-ablative Nd:YAG laser [12] and one case report the fractional ablative Er:YAG laser [8]. Laser therapy improves symptoms in most studies [13]. However, methodology and results were too heterogeneous, patients too few and follow-up periods too short for a recommendation [13].

Fractional ablative CO_2_ laser therapy (10′600 nm) sets visible, possibly painful micro-wounds by superficial skin evaporation [7] and makes thin channels into the tissue to facilitate temperature penetration. Heat stimulates connective tissue remodeling, collagen production, and re-vascularization [14]. In contrast, the non-ablative Nd:YAG laser (1064 nm) has very low absorption in water and thus, penetrates deeply into tissue. Long Nd:YAG laser pulses do not damage the skin surface, but generate a slow gentle temperature increase in deeper lying structures (> 5 mm) and induce the heat response [12, 15, 16]. Two RCTs demonstrated significant between-group and in-group improvement favoring the CO_2_ or Nd:YAG lasers over the steroid treatment [11, 12]. The third RCT compared CO_2_ laser vs. sham treatment, and found no significant between-group difference in histopathologic change [7]. Long-term and high-quality RCTs are needed before laser can be considered a routine treatment for LS [10, 13].

The objective of this study was to assess efficacy, safety, and sustainability of a novel dual Nd:YAG/Er:YAG laser treatment concept for LS. The heat generated by the Nd:YAG laser is expected to penetrate through hyperkeratosis, epithelium, and subepithelial sclerosis, reaching the deeper regenerative tissue area to reduce inflammation and induce collagen remodeling and neovascularization. Additionally, the ablative Er:YAG laser is expected to reduce superficial hyperkeratosis and other skin irregularities. The study was designed as a prospective, single center, investigator-initiated RCT, using topical steroid, the current gold standard, as the comparator. The study hypothesis is that the Nd:YAG/Er:YAG laser combination is effective and can achieve similar results as the standard steroid treatment.

## Methods

### Design

This study is a post-market, single center, prospective, randomized, active-controlled investigator-initiated clinical trial. It was performed at an ambulatory tertiary referral center for bladder, vulvar, and pelvic floor disorders at the Department of Gynecology and Obstetrics, Cantonal Hospital Frauenfeld, Switzerland. All women with clinical presentation of LS were asked to participate. The efficacy and safety of a novel dual Nd:YAG/Er:YAG laser (FotonaSmooth SP® Spectro laser (Model M021-4AF/3)) were compared to the gold standard therapy with topical steroids to treat vulvar LS. The study followed the Declaration of Helsinki, received ethical approval (EKOS 19/056, BASEC-ID: 2019–00,634), and was registered at clinicaltrials.gov (NCT03926299).

### Inclusion criteria at time of randomization

1. Woman age 18 or older.
2. Clinical diagnosis for LS (chronic pruritus of non-fungal origin, burning sensations, soreness, and dyspareunia, no oral lesions).
3. Clinical LS score ≥ 4 [4, 17].
4. Agreement for two vulvar punch biopsies before and after treatment.

### Exclusion criteria at time of randomization

1. Concomitant steroid, calcineurin inhibitor or any other topical or systemic treatment for LS (therapy has to be stopped ≥ 2 weeks before the screening visit).
2. < 3 months since start of vaginal estrogen treatment.
3. Malignant disease as the cause of the symptoms, including precursors, e.g., differentiated exophytic vulvar intraepithelial lesion or vulvar acanthosis with altered differentiation.
4. BMI > 35 kg/m^2^.
5. Acute infection (fungal, bacterial, viral) of the vulva, vagina or bladder.
6. Presence of contraindications for the laser treatment or topical steroid treatment (e.g., using drugs causing photosensitivity or a hypersensitivity/allergy to clobetasol propionate)
7. ≤ 3 months since labor, miscarriage or an operation in the lower abdomen.
8. Pregnancy, breast feeding or the intention to become pregnant during the study.
9. Lack of safe contraception for the study duration.
10. Participation in another study with investigational drug within the 30 days preceding or during the present study.
11. Enrollment of the investigator, his/her family members, employees and other dependent persons.
12. Unwillingness or inability to comply with study plan.
13. Unwillingness or inability to consent.

### Withdrawal management

Participants with one of the following criteria were allowed or required to withdraw from the study:

1. Voluntary withdrawal at any time without giving any reason.
2. Withdrawal by the investigator if it was in the best interest for the patient, e.g., after an adverse event such as the diagnosis of a disease, or when her safety was at risk.
3. Non-compliance to the study protocol.

If a patient had withdrawn before completion of the laser or the steroid therapy, she was replaced.

## Protocol

### Study plan

At the screening visit, eligibility was evaluated, patients gave informed consent. Randomization and allocation were done at baseline. The laser intervention group received four laser treatments, the comparator group received topical steroids for 6 months. Follow-up visits were at 6, 12, and 24 months after baseline. Vulvar biopsies were taken at the screening visit and at the 6-month follow-up (Table 1). Steroid therapy had to be stopped ≥ 2 weeks before the first biopsy.

**Table 1:** Study plan, treatment of LS, RCT laser vs. steroid

	Enrollment	Post-allocation						Follow-ups		
TIMEPOINT	Screening -1 M^a^	Baseline 0 M	1 M^b^	2 M	(3 M)^c^	4 M	(5 M)^c^	6 M	12 M	24 M^d^
VISITS:	X	X	(X)	X		X		X	X	X
ENROLLMENT:										
Eligibility screen:	X									
Demographics, medical history	X									
Inclusion/exclusion criteria	X									
Informed consent	X									
Allocation		X								
INTERVENTIONS:										
Laser treatment:		X	X	X		X				
VAS pain during/after laser treatment		X	X	X		X				
Steroid treatment^e^:										
Hand out steroid medication and treatment scheme		X		X		X				
ASSESSMENTS:										
Clinical examination, photo documentation and sketch	X	X	(X)	X		X		X	X	
Clinical LS score	X	X	(X)	X		X		X	X	
VSQ	X	X	(X)	X		X		X	X	X
VAS symptom strength	X	X	(X)	X		X		X	X	X
Adverse events		X	(X)	X		X		X	X	X
PGI-I								X	X	X
Therapy log		X	(X)	X		X		X	X	X
Treatment history									X	X
Telephone interview										X
Vulvar biopsy	X							X		

*M* month, *VAS* visual analog scale (0–10), *LS* lichen sclerosus, *VSQ* Vulvovaginal Symptoms Questionnaire, *PGI-I* Patient Global Impression of Improvement

^a^Any concomitant steroid treatment has to be stopped ≥ 2 weeks before

^b^1 M visit and assessment only for laser group (X)

^c^no visits at (3 M) and (5 M)

^d^telephone visit

^e^steroid dose: high (months 1 and 2), medium (months 3 and 4), low (months 5 and 6)

### Description of the intervention

#### Laser arm

The Nd:YAG/Er:YAG laser therapy was applied four times in an ambulatory setting: at baseline and after 1, 2, and 4 months (Table 1). The laser settings are shown in Table 2. One laser session takes 10–15 min. Topical lidocaine cream is optional and can be applied when needed.

**Table 2:** Laser settings for the treatment of LS

	Step 1	Step 2
Laser^a^	Nd:YAG (1064 nm)	Er:YAG (2940 nm)
Handpiece	R33	R11
Mode	PIANO	MSP
Fluence^b^	90 J/cm^2^ (70–100 J/cm^2^)	Ultra-light peel: 1 J/cm^2^ Light peel: 3 J/cm^2^
Penetration into the skin	Deep (> 5 mm)	Superficial (< 20 μm)
Frequency	NA	2 Hz
Pulse width	5 s pulse (0.3 s pause)	100 μs pulse
Spot size	9 mm	5 mm
Passes	5 passes in a brushing mode	1–2 passes on vulvar areas with hyperkeratosis and erosions
Effect on the tissue	Non-ablative, thermal effect on the tissue inhibits inflammation and stimulates tissue regeneration	Ablation removes erosions and hyperkeratosis/leukoplakia
Treatment schedule	4 sessions: at baseline, and 1, 2 and 4 months after baseline	Immediately following step 1

^a^Laser settings can be set in the “Expert mode”

^b^Adjust fluence depending on tolerability. Begin with a low fluence and increase in subsequent laser sessions. Optional application of local lidocaine cream before laser session

#### Steroid arm

Topical steroid (clobetasol 0.05%) cream was applied as the active control treatment for 6 months. Patients started with a high dose which subsequently was reduced after 2 and 4 months (Table 1), following this treatment regime*:

- Phase 1 (first and second month): at four evenings of the week, every week.
- Phase 2 (third and fourth month): at four evenings of the week, every other week.
- Phase 3 (fifth and sixth month): at four evenings of the week, first week of the month.

*modified from the University Hospital Zurich scheme [18].

The treatment was complemented with a moisturizing cream twice a day (morning and evening), except on the evenings when steroid cream was applied. Treatment success depends on the patients’ compliance.

### Primary outcome

The primary outcome of this study was to assess the efficacy of the Nd:YAG/Er:YAG laser combination therapy to treat LS by evaluating the change of the LS score from baseline to the 6-month follow-up. The LS score is an objective, validated, physician-administered clinical score to measure the severity of vulvar LS [4, 7, 19]. A physician assesses each of the six criteria (1) erosions, (2) hyperkeratosis, (3) fissures, (4) agglutination, (5) stenosis, and (6) atrophy on a scale from 0 (normal/none), 1 (a few signs/moderate) to 2 (clear signs/severe) [17]. Hence, the LS score ranges from 0 to 12. An LS score ≥ 4 identifies LS with a probability of > 90% [4]. Individual characteristics for “none”, “moderate”, or “severe” are defined [4].

### Secondary outcomes

Secondary outcomes were assessed as comparisons between baseline and follow-ups within the laser or the steroid group, and as comparisons between laser vs. steroid group. The following measurements were evaluated:

- Demographics and medical history.
- Clinical examination, photo documentation, and clinical sketch of the vulva to localize symptoms, biopsy, and laser treatment.
- Objective clinical LS score [4, 17].
- Subjective Vulvovaginal Symptoms Questionnaire (VSQ) [20], a validated evaluation of the four domains “symptoms” (seven questions), “emotions” (four questions), “life impact” (four questions), and “sexual impact” (five questions, four of them for sexually active women). The score ranges from 0 (no symptoms) to 16 or 20 for sexually active women (severe symptoms).
- Subjective evaluation of symptom strength (VAS 0-10) of the four typical symptoms: vulvar itching, vulvar burning or stinging, vulvar pain and dyspareunia.
- Patient satisfaction by the Patient Global Impression of Improvement (PGI-I) questionnaire [21].
- Discomfort/pain (VAS 0–10) and adverse events during and immediately after each laser session.
- Total joule counts and number of pulses per laser session.
- Device deficiencies.
- Treatment history between visits.
- Adverse events. Patients are asked systematically at each visit. Documentation includes dates of event, treatment, resolution, assessment of seriousness and causal relationship to device and/or study procedure.
- Histopathological evaluation of vulvar biopsies by an external, independent expert pathologist.

### Numbers

Sample size calculation was performed for the primary endpoint, the clinical LS score at 6 months. A total of 34 patients in the laser arm would provide 80% power to detect a medium effect size of 0.5 [22] with a two-sided alpha set at 5%. Assuming a drop-out rate of 10–15%, 40 patients were needed in the laser arm.

For a superiority/inferiority test between groups, a total sample size of 60 patients—40 in the laser group, 20 in the steroid group—would provide 80% power to detect a large effect size of 0.8 [22] with a two-sided alpha set at 5%. Adding again a buffer of 10% for drop-outs, we aimed to recruit 66 patients, 44 in the laser group and 22 in the steroid group.

### Randomization

At baseline, the study participants were randomly allocated to the laser or the steroid arm in a 2:1 ratio. Stratification was done according to the severity of the clinical LS score: clinical LS score 4, 5 or 6 (LSL low) vs. LS score > 6 (LSH high). Block randomization was applied for each stratum. An independent scientist without patient contact prepared the randomization process. Study doctors got sealed, consecutively labeled envelopes for each stratum. Envelopes were opened in subsequent order at baseline before the first treatment. The allocation to the treatment arm was noted on the case report form at baseline. Blinding of patients for the allocated intervention was not possible. Two persons independently scored the clinical findings. The investigating pathologist was blinded for allocation.

### Statistical methods

Baseline comparison of the two treatment arms is assessed by one-way ANOVA or *t *test (for continuous variables), Poisson regression (for count data) or Fisher test (for categorical or binomial data). If the assumption of normal distribution is violated, non-parametric alternatives (Friedman, Kruskal–Wallis, and Wilcoxon Signed-Rank tests) can be used for the analysis. For all tests, a significance level of alpha = 5% is applied.

The analysis of changes to baseline is done with the *t *test, testing the null hypothesis that there is no difference between baseline and follow-ups. Further analysis is done testing the null hypothesis that there are no differences between the two treatment arms (*t* test).

For subgroup analysis, mean differences between baseline and follow-ups are stratified according to the subgroup findings at baseline. The interaction effect of baseline characteristics on treatment effect is investigated by linear regression.

### Follow-ups

Follow-up visits were at 6 and 12 months after baseline, and a telephone interview was planned at 24 months. At 6 months, the primary outcome was evaluated and a second vulvar biopsy was taken (Table 1). Between 6 and 12 months, patients in the steroid arm were offered four optional laser treatments, patients in the laser arm could optionally request further laser treatments, and patients in both arms were allowed to optionally use topical steroids. At 12 months, all study parameters including the treatment history between 6 and 12 months were registered. At the telephone interview at 24 months, subjective parameters and the treatment history between 12 and 24 months were recorded.

## Discussion

Today, laser therapy is propagated as a solution for a variety of indications, including gynecological diseases. Everybody uses the laser. However, based on the actual data situation, experts cannot give recommendations yet, but request more studies, ideally RCTs with longer observation periods [10, 23, 24].

The presented study was designed as an RCT with a long follow-up period to evaluate a novel laser treatment for LS. In a standardized research setting, the study will give answers regarding tolerability and safety of a new non-invasive double laser concept for the treatment of vulvar LS, efficacy and durability of the laser treatment with an observation time of 2 years, patient satisfaction and therapy adherence, effect of the laser treatment on the physician-evaluated clinical outcome in comparison to subjective symptom changes and improvement of quality of life, efficacy of the laser treatment in comparison to the current gold standard with topical steroid treatment, identification of patient groups best suited for laser treatment, and histopathological changes after laser and after steroid therapy.

The study design aimed to minimize the drop-out ratio. It may help that the patients in both study arms receive an active therapy rather than a sham/placebo treatment. Suffering should also be minimized by allowing the use of any symptom relieve after the 6-month visit. Frequent study visits for both study arms further help to keep patients involved, and the prospect of laser treatment after 6 months encourage patients in the steroid arm to comply with the study setting.

The relatively low patient number at just one trial center can be interpreted as a trial limitation. However, the suggested treatment is new and must be tested before upscaling. Furthermore, this study setting allows a uniform patient inclusion, treatment, and data acquisition and is expected to deliver consistent data.

The results of this study are highly expected from numerous practitioners and—above all—from the many patients with a long disease history. The new double Nd:YAG/Er:YAG laser treatment is minimally invasive, free of steroids, and requires only four interventions. Furthermore, the RCT setting will show whether the dual laser combination can be an alternative to the standard steroid treatment.
