# Correction: Nd:YAG/Er:YAG dual laser vs. topical steroid to treat vulvar lichen sclerosus: study protocol of a randomized controlled trial

**DOI:** 10.1007/s00404-023-07162-x

**Published:** 2023-10-24

**Authors:** Volker Viereck, Marianne Gamper, Sigrid Regauer, Claudia Walser, Irena Zivanovic

**Affiliations:** 1 Department of Gynecology and Obstetrics, Cantonal Hospital Frauenfeld Postfach, 8501, Frauenfeld, Switzerland; 2 Institute of Pathology, Medical University Graz, Graz, Austria

**Correction: Archives of Gynecology and Obstetrics (2023) 308:643–649** 10.1007/s00404-023-07055-z

Table 1 of this article was mistakenly published with incorrect data. The correct Table 1 is provided below.

The original article is also updated.

**Table 1:**
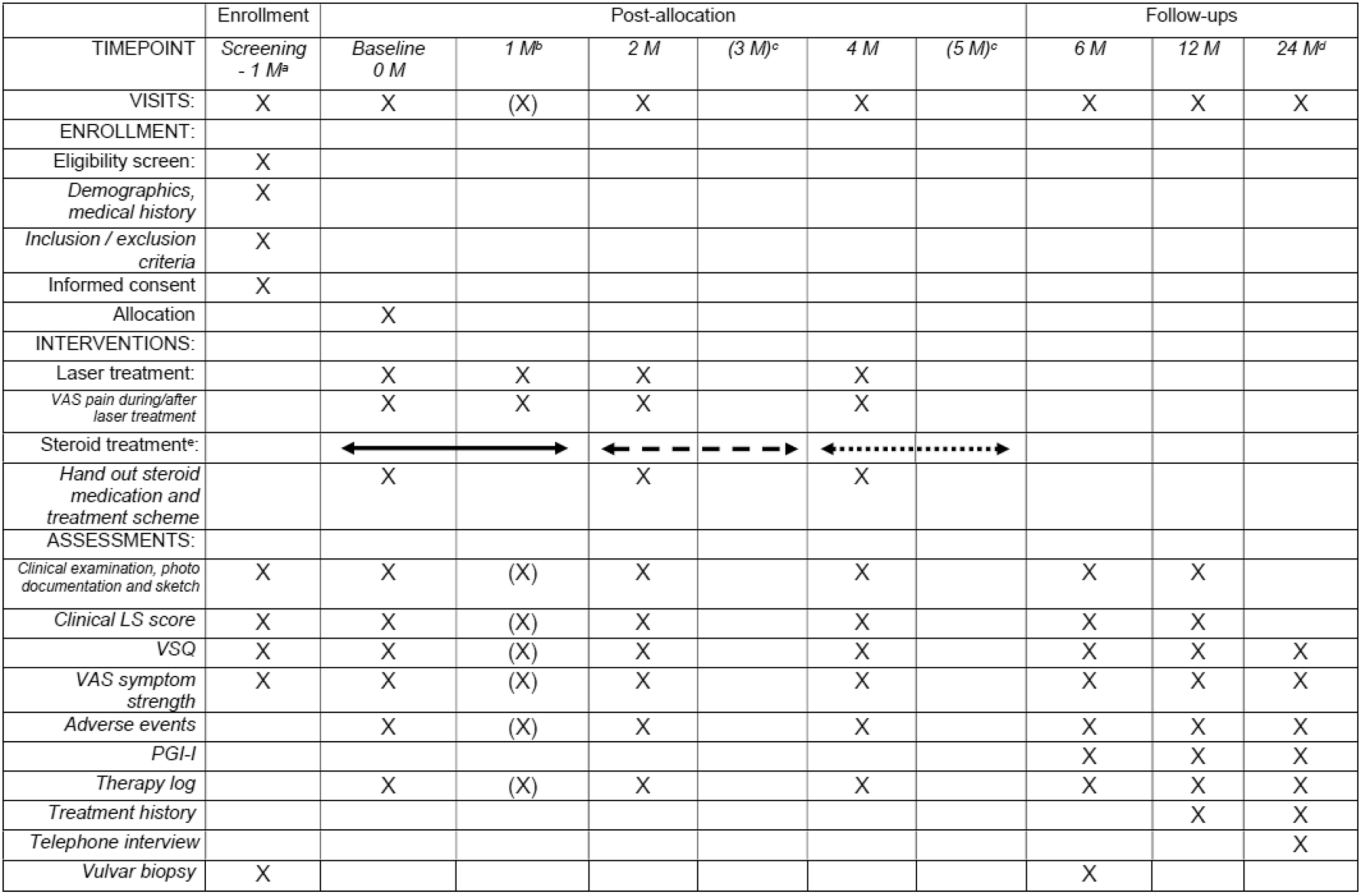
Study plan, treatment of LS, RCT laser vs. steroid

*M* month, *VAS* visual analog scale (0–10), *LS* lichen sclerosus, *VSQ* Vulvovaginal Symptoms Questionnaire, *PGI-I* Patient Global Impression of Improvement

^a^Any concomitant steroid treatment has to be stopped ≥ 2 weeks before

^b^1 M visit and assessment only for laser group (X)

^c^No visits at (3 M) and (5 M)

^d^Telephone visit

^e^Steroid dose: high (months 1 and 2), medium (months 3 and 4), low (months 5 and 6)

